# 1,2,3-Triazole-Containing Compounds as Anti–Lung Cancer Agents: Current Developments, Mechanisms of Action, and Structure–Activity Relationship

**DOI:** 10.3389/fphar.2021.661173

**Published:** 2021-06-11

**Authors:** Ting Liang, Xiangyang Sun, Wenhong Li, Guihua Hou, Feng Gao

**Affiliations:** ^1^ Key Laboratory for Experimental Teratology of the Ministry of Education and Biomedical Isotope Research Center, School of Basic Medical Sciences, Cheeloo College of Medicine, Shandong University, Jinan, China; ^2^ Department of Interventional Radiology, Qilu Hospital of Shandong University, Jinan, China; ^3^ Department of Oncology, Shandong Provincial Hospital Affiliated to Shandong First Medical University, Jinan, China

**Keywords:** hybrid molecules, lung cancer, anticancer, structure–activity relationship, triazole

## Abstract

Lung cancer is the most common malignancy and leads to around one-quarter of all cancer deaths. Great advances have been achieved in the treatment of lung cancer with novel anticancer agents and improved technology. However, morbidity and mortality rates remain extremely high, calling for an urgent need to develop novel anti–lung cancer agents. 1,2,3-Triazole could be readily interact with diverse enzymes and receptors in organisms through weak interaction. 1,2,3-Triazole can not only be acted as a linker to tether different pharmacophores but also serve as a pharmacophore. This review aims to summarize the recent advances in 1,2,3-triazole–containing compounds with anti–lung cancer potential, and their structure–activity relationship (SAR) together with mechanisms of action is also discussed to pave the way for the further rational development of novel anti–lung cancer candidates.

## Introduction

Lung cancer has high morbidity and represents the leading cause of cancer-related deaths (around 20% of all mortalities in cancer) (Nasim et al., 2019; Willis et al., 2019; Bade and Dela Cruz, 2020; Majem et al., 2020; Salehi et al., 2020; Zhang et al., 2020). With the development of novel anticancer agents and improvement of technology, lung cancer treatment has achieved great progress in recent decades, but the morbidity and mortality rates remain high and the overall 5-year survival rate is only around 15% (Gray et al., 2019; Coakley and Popat, 2020).

**FIGURE 1:**
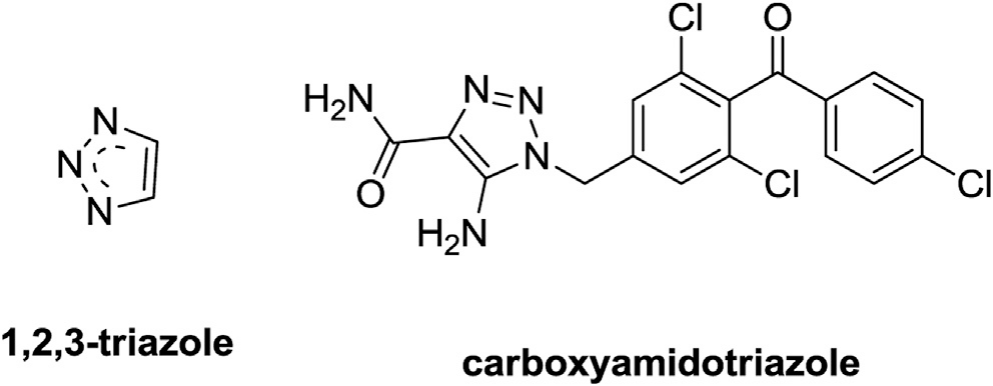
Chemical structures of 1,2,3-triazole moiety and carboxyamidotriazole.

1,2,3-Triazole moiety (Figure 1), a major pharmacophore system among nitrogen-containing heterocycles, can be obtained readily using “click” chemistry with copper- or ruthenium-catalyzed azide-alkyne cycloaddition reactions and it can also act as a linker to tether different pharmacophores. Moreover, 1,2,3-triazole could form diverse non-covalent interactions, such as hydrogen bonds, van der Waals forces, and dipole–dipole bonds with various enzymes, proteins, and receptors (Bonandi et al., 2017; Bozorov et al., 2019). Hence, 1,2,3-triazole derivatives exhibit a variety of promising biological properties, including antibacterial (Zhang, 2019; Xu, 2020), antimalarial (Chu et al., 2019; Feng et al., 2020), antitubercular (Zhang et al., 2017; Yan et al., 2020), antiviral (Rani et al., 2020; Feng et al., 2021), and anticancer (Lal and Yadav, 2018; Slavova et al., 2020) activities. For example, 1,2,3-triazoles could exert the anticancer potential by inducing the cell cycle arrest and apoptosis of cancer cells. The 1,2,3-triazole–containing agent carboxyamidotriazole (CAI) could synergise with sorafenib to combat NSCLC by inhibiting NANOG and aggravating apoptosis (Ferreira et al., 2013; Chen et al., 2017). These studies suggest that 1,2,3-triazole derivatives are useful scaffolds for development of novel anti–lung cancer agents with low toxicity and high efficacy.

In recent years, a variety of 1,2,3-triazole–containing compounds are designed, synthesized, and evaluated for their anti–lung cancer activity, and some of them have the potential to act on dual-/multi-targets simultaneously (Jain and Piplani, 2019; Xu et al., 2019; Farrer and Griffith, 2020; Sahu et al., 2020). This review summarizes the current developments, mechanisms of action, and structure–activity relationships (SARs) of 1,2,3-triazole–containing compounds with anti–lung cancer potential to pave the way for further rational development.

## 1,2,3-Triazole–Containing Chromene/Coumarin Derivatives

Chromene (benzopyran) and coumarin (chromen-2-one) moieties are present in a large number of biologically active compounds, and their derivatives are potent inhibitors of a variety of proteins, such as EGFR, tyrosine kinase, ERK1/2, PI3K, HSP 90, Bax, STAT proteins, nuclear factor kappa-light-chain-enhancer of activated B cells (NF-κB), and telomerase associated with lung cancer (Manjinder et al., 2015; Manvendra et al., 2018; Al-Warhi et al., 2020; Oliveira-Pinto et al., 2020). Therefore, therapeutic drug candidates could be obtained by combination of 1,2,3-triazole and chromene/coumarin for the treatment of lung cancer, even drug-resistant and multidrug-resistant (MDR) forms.

**FIGURE 2:**
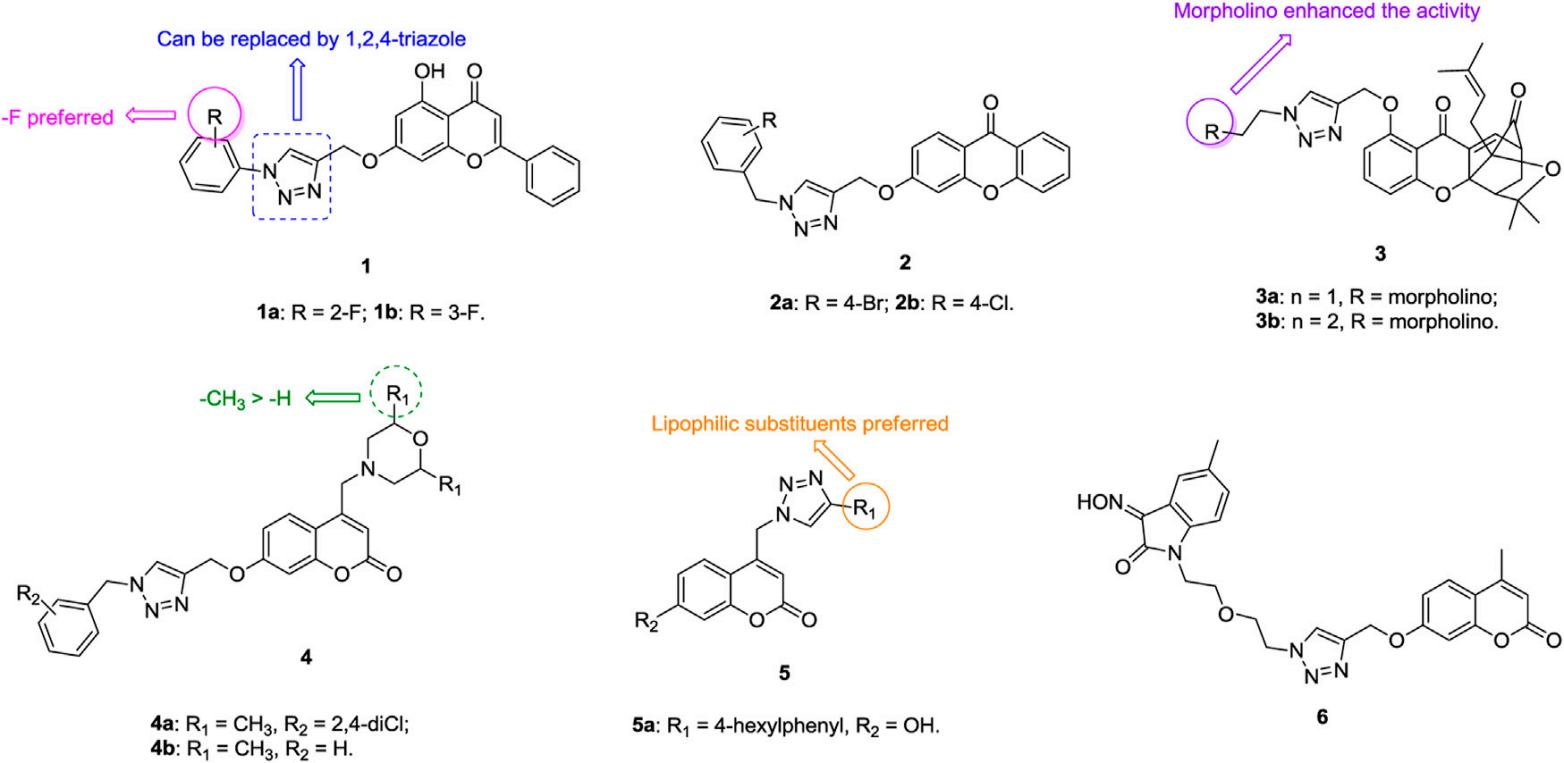
Chemical structures of 1,2,3-triazole–containing chromene/coumarin derivatives one to six.

1,2,3-Triazole–containing chromene derivatives **1** (Figure 2) possess broad-spectrum activity against six human cancer cell lines, and an antiproliferative SAR study against the A549 lung cancer cell line (IC_50_: 1.02–74.28 μm, MTT assay) revealed that the fluoro atom on the phenyl ring contributes to the activity. However, 1,2,3-triazole is not crucial for the activity and replacement of 1,2,3-triazole by 1,2,4-triazole is also tolerated (Luan et al., 2020). In addition, the phenyl group at the C-2 position of the chromene moiety has great influence on the activity, as evidenced by the decreased activity of compounds **2** (IC_50_: 32.4–596.6 μm, MTT assay) against A549 cells (Wu et al., 2019). Among them, compounds **1a,b** (IC_50_: 27.89 μm), the most active against A549 cells, are less potent than the reference Chrysin (IC_50_: 8.80 μm).

1,2,3-Triazole–containing chromene derivatives **3** (IC_50_: 0.28–6.30 μm, MTT assay) exhibit excellent activity against A549, taxol-resistant A549 (A549/taxol), and cisplatin-resistant A549 (A549/cisplatin) cancer cell lines, and an SAR study proved that the morpholino group at the R position could enhance the activity (Li et al., 2017). In particular, compound **3a** (DDO-6318, IC_50_: 0.31–0.42 μm) is highly potent against A549, A549/taxol and A549/cisplatin lung cancer cell lines, and the resistance index (RI: IC_resistant cells_/IC_sensitive cells_) is approximately 1, demonstrating its potential for the treatment of drug-sensitive and drug-resistant lung cancers. The activity of DDO-6318 is at the same level as those of DDO-6106 (IC_50_: 0.46 μm) and gambogic acid (IC_50_: 0.29 μm) against A549/taxol cells but is around eight times superior to those of DDO-6106 (IC_50_: 0.46 μm) and gambogic acid (IC_50_: 0.29 μm) against A549 and A549/cisplatin cells. In the mouse model xenografted with A549, DDO-6318 (71.32% inhibition in tumor growth at 20 mg/kg, twice daily doses/BID, and intravenous administration) displays significant inhibitory effect on the growth of inoculated A549 in mice without causing vascular irritation or weight loss, and its activity is higher than those of DDO-6101 (34.56% inhibition at 20 mg/kg) and 5-fluorouracil (64.71% inhibition at 20 mg/kg). Similar results were also observed when oral administraion of DDO-6318 (66.43% inhibition at 50 mg/kg daily oral dose for DDO-6318 and 21.43% inhibition for DDO-6101at the same dose). Thus, DDO-6318 can be considered as a new and orally active natural product–like anti–lung cancer candidate for further clinical studies.

Some 1,2,3-triazole–containing coumarin derivatives **4** (IC_50_: 0.8–27.08 μm, MTT assay) are active against various cancer cell lines, and an antiproliferative SAR study against A549 cells illustrates that the methyl group at the R_1_ position could result in the improved activity (Liu et al., 2017; Goud et al., 2019). Among them, compounds **4a,b** (IC_50_: 2.97 and 4.78 μm) are more potent than cisplatin (IC_50_: 24.15 μm) against A549 cells. Moreover, compound **4a** (IC_50_: 49.07 μm) shows low cytotoxicity toward mouse embryonic fibroblast cells NIH/3T3, and the selective index (SI: IC_50(NIH-3T3)_/IC_50(A549)_) is 16.5. A mechanistic study elucidated that compound **4a** could induce sub-G1 phase arrest, increase apoptosis, decrease the mitochondrial membrane potential, and promote reactive oxygen species (ROS) production. 1,2,3-Triazole–containing coumarin derivatives **5** (IC_50_: 8.73->100 μm, MTT assay) show weak to moderate activity against a panel of cancer cell lines, including A549 cells, and a strong correlation exists between lipophilicity profile and antiproliferative activity (Kraljevic et al., 2016). Compound **5a** (IC_50_: 8.87 μm) displays the highest inhibitory activity against A549 cells, but it is toxic against normal fibroblasts WI38 (IC_50_: 13.96 μm).

Generally, incorporation of the third fragment, such as β-lactam (Dhawan et al., 2020) and uracil (Sanduja et al., 2020), is detrimental to the antiproliferative activity against lung cancer cells, but some 1,2,3-triazole–tethered coumarin-isatin hybrids, such as compound **6** (IC_50_: 18.67 μm, SRB assay), show certain activity against A549 cells (Diao et al., 2019; Xu et al., 2019). Hence, these compounds still need further modification.

## 1,2,3-Triazole–Containing Chalcone Derivatives

Chalcones are scaffolds that appear as an important structural component in various natural products and possess some useful biological properties. Chalcones are potential inhibitors of aromatase, P-glycoprotein (P-gp), histone deacetylase (HDAC), matrix metalloproteinase (MMP), NF-κB, tubulin, vascular endothelial growth factor, and vascular endothelial growth factor receptor 2 (VEGFR-2) kinase; thus, chalcones are endowed with broad-spectrum antiproliferative activity against drug-susceptible and drug-resistant cancers, even MDR cancers (Karthikeyan et al., 2015; Gao et al., 2020). Accordingly, 1,2,3-triazole-–containing chalcone derivatives are useful prototypes for the discovery of novel anticancer candidates.

**FIGURE 3:**
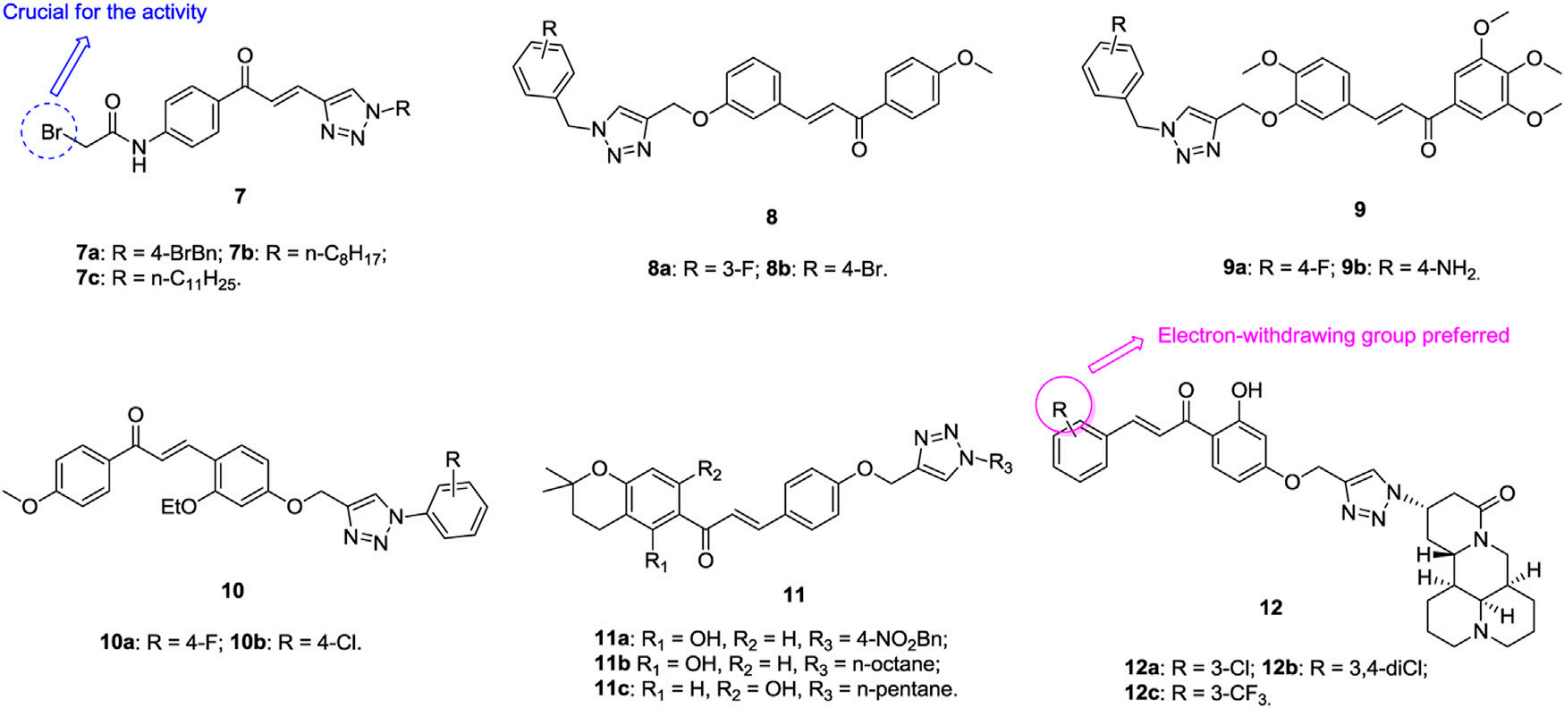
Chemical structures of 1,2,3-triazole–containing chalcone derivatives 7–12.

1,2,3-Triazole–containing chalcone derivatives **7** (Figure 3, IC_50_: 8.67–11.62 *μ*M, MTT assay) show potential activity against A549 cells. An SAR study illustrated that the bromo group is essential for the activity, and replacement by heterocycles results in loss of activity (Vanaparthi et al., 2020). Among them, compounds **7a,c** (IC_50_: 8.67 and 9.74 μm) with single digital micromolar inhibitory activity are comparable to doxorubicin (IC_50_: 3.24 μm). Compounds **8** (IC_50_: 9–60 μm, MTT assay) are active against A549 cells, and a mechanistic study revealed that these compounds could exert antiproliferative activity by inducing apoptosis and G2/S arrest as well as triggering mitochondrial potential loss (Yadav et al., 2017). An SAR study demonstrated that introducing a methoxy group into the phenyl ring could not increase the activity apparently, and the activity of compounds **9** (GI_50_: 4.7–11.9 μm, SRB assay) is considerably inferior to that of combretastatin-A4 (GI_50_: 0.08 μm) against A549 cells (Hussaini et al., 2016). Further study proved that the benzyl group at the N-1 position of the 1,2,3-triazole moiety is not crucial for the activity, and replacement by the phenyl ring is also permitted, as evidenced by that compounds **10a,b** (IC_50_: 62.51 and 75.41 μm, MTT assay) show comparable activity to doxorubicin (IC_50_: 39.86 μm) against A549 cells (Raghavender et al., 2020). The 1,2,3-triazole–containing chalcone compounds **11a**-**c** (IC_50_: 35.81–50.82 μm, MTT assay) are more potent than doxorubicin (IC_50_: 69.33 μm) against A549 cells, demonstrating their potential to fight against lung cancer (Chinthala et al., 2015).

The antiproliferative SAR of 1,2,3-triazole–tethered chalcone-matrine hybrids **12a-c** (IC_50_: 5.01–12.72 μm, MTT assay) against A549 cells reveals that the electron-withdrawing group at the R position could boost up the activity, and the representative compound **12a** (IC_50_: 5.01–7.31 μm) not only exhibits the highest activity against the four tested cancer cell lines but also displays low cytotoxicity (IC_50_: 39.21 μm) toward normal NIH/3T3 cells (Zhao et al., 2015). A mechanistic study reveals that compound **12a** could induce the apoptosis of A549 cells concentration-dependently in the A549-xenografted nude mouse model, and it also (10 mg/kg, tail vein injection) shows 85.4% tumor growth inhibition (TGI) after treatment for 16 days without causing obvious toxicities. Accordingly, compound **12a** could be considered as a promising candidate for the chemotherapy of lung cancer.

## 1,2,3-Triazole–Containing Indole Derivatives

Indoles, which could inhibit various enzymes and receptors, such as HDAC, proviral insertion site in moloney murine leukemia virus (Pim), and tubulin in cancer cells, are scaffolds to avail for developing novel anticancer agents (Guo and Diao, 2020; Xu and Xu, 2020). Thus, combination of 1,2,3-triazole with indole represents a promising strategy to develop novel anticancer candidates that are effective against drug-sensitive and drug-resistant cancers.

**FIGURE 4:**
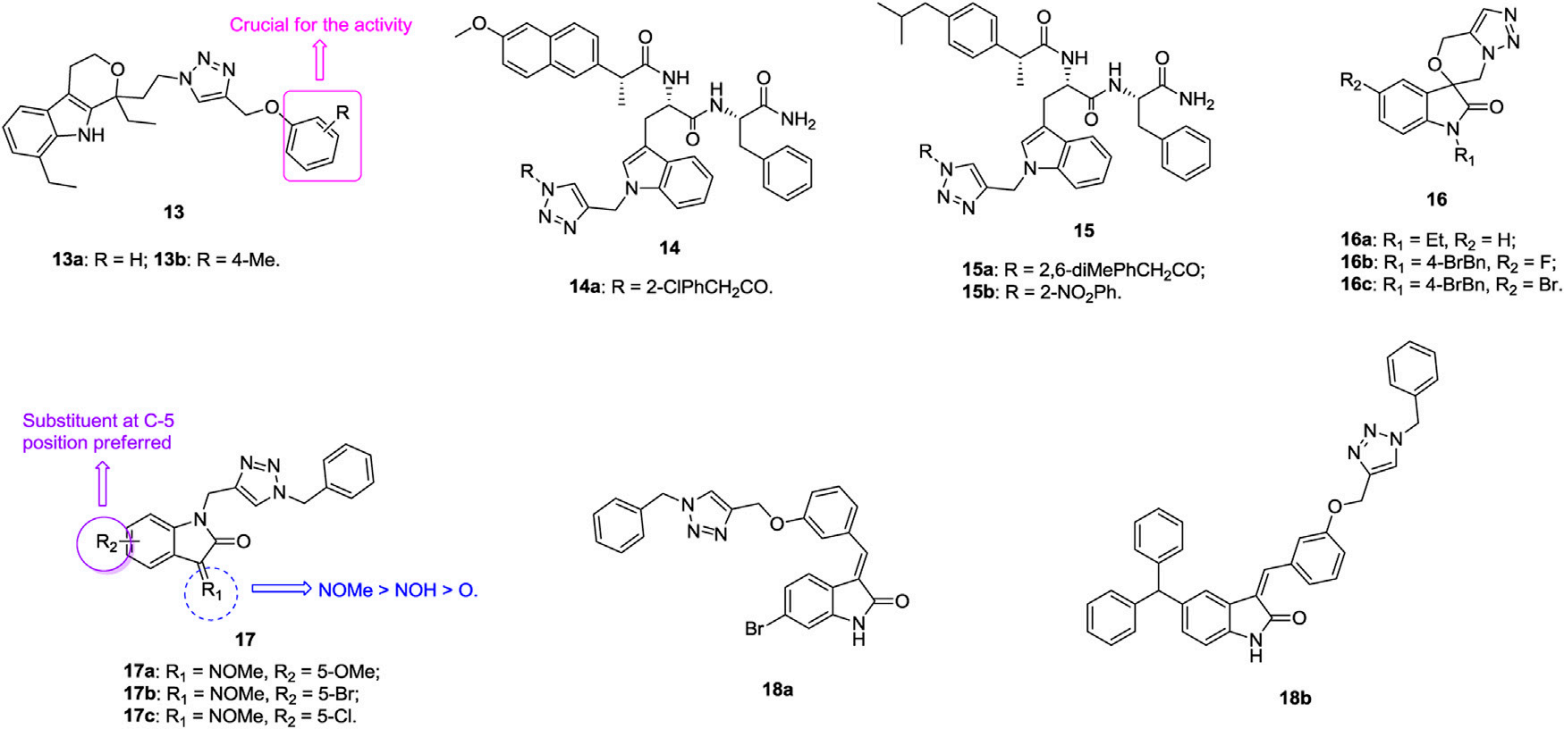
Chemical structures of 1,2,3-triazole–containing indole derivatives 13–18.

An antiproliferative SAR study of 1,2,3-triazole–containing etodolac derivatives **13** (Figure 4, IC_50_: 3.29–10.71 μm, MTT assay) against A549 cells revealed that the phenyl ring is crucial for the activity, and replacement by the naphthyl group results in loss of activity (Kummari et al., 2017). Introduction of substituents into the phenyl ring is generally detrimental to the activity, whereas methyl at the *para* position could enhance the activity slightly. The most active compounds **13a,b** (IC_50_: 3.65 and 3.29 μm) display comparable activity to doxorubicin (IC_50_: 3.30 μm). Thus, these compounds can serve as promising lead candidates for further investigations. 1,2,3-Triazole–containing indole derivatives **14** (IC_50_: 9.07–47.11 μm, MTT assay) and **15** (IC_50_: 15.25–38.68 μm, MTT assay) show considerable activity against A549 cells, and the most potent compound **14a** (IC_50_: 9.07 μm) is not inferior to doxorubicin (IC_50_: 4.39 μm) (Srinivas et al., 2020; Suryapeta et al., 2020).

The majority of spirooxindole-derived morpholine-fused 1,2,3-triazoles **16** (IC_50_: 1.87–23.44 μm, MTT assay) are active against A549 cells, and an SAR study implied that the *para* bromobenzyl group is more favorable than the alkyl group at the N-1 position of oxindole moiety (Senwar et al., 2015). In particular, the activity of compound **16a** (IC_50_: 1.87 μm) is comparable to that of doxorubicin (IC_50_: 1.98 μm) but 3.8-fold superior to that of 5-fluorouracil (IC_50_: 7.24 μm). Further investigations demonstrated that compound **16a** could arrest the A549 cells at the G2/M phase of the cell cycle and induce apoptosis in A549 cells through collapse of the mitochondrial membrane potential as well as elevation of intracellular ROS levels. Overall, compound **16a** has the potential to act as a lead compound for the development of novel anticancer agents against lung adenocarcinoma A549 cancer cells.

An antiproliferative SAR study of 1,2,3-triazole–containing isatin compounds **17** (IC_50_: 0.99–27.33 μm, MTT assay) against NCI-H23 lung cancer cells disclosed that substituents at the C-3 and C-5 positions have great influence on the activity (Lan et al., 2020). Introduction of oxime to the C-3 position could increase the activity, and the relative contribution order is methoxime > oxime > ketone. Electron-donating methoxy and halogen atoms at the C-5 position are preferred, whereas movement to the C-7 position causes great loss of activity. In particular, the most active compound **17a** (IC_50_: 0.65 μm, MTT assay) is around 2 and 13 times superior to doxorubicin (IC_50_: 1.12 μm) and 5-fluorouracil (IC_50_: 8.84 μm), respectively. Some other 1,2,3-triazole–containing isatin compounds also hold certain activity against A549 cells, and the representative compounds **18a,b** (IC_50_: 14.7 and 9.6 μm, MTT assay) demonstrate the highest activity (Sharma et al., 2015; Nagarsenkar et al., 2016; Fan et al., 2018; Xu et al., 2018). Mechanistic studies indicate that hybrid **18a** could induce cell apoptosis, cause cell cycle arrest at the G2/M phase, and lead to collapse of the mitochondrial membrane potential (Nagarsenkar et al., 2016).

## 1,2,3-Triazole–Containing Podophyllotoxin/Epipodophyllotoxin Derivatives

Podophyllotoxins show antiproliferative activity by inhibiting tubulin polymerization, and epipodophyllotoxins are inhibitors of topoisomerase II (Sathish et al., 2018; Xiao et al., 2020). Some podophyllotoxin-/epipodophyllotoxin-based agents, such as etoposide and teniposide, have already been approved for cancer therapy, revealing that podophyllotoxin/epipodophyllotoxin derivatives are promising as novel anticancer agents (Kamal et al., 2015; Routh and Nandagopal, 2017).

**FIGURE 5:**
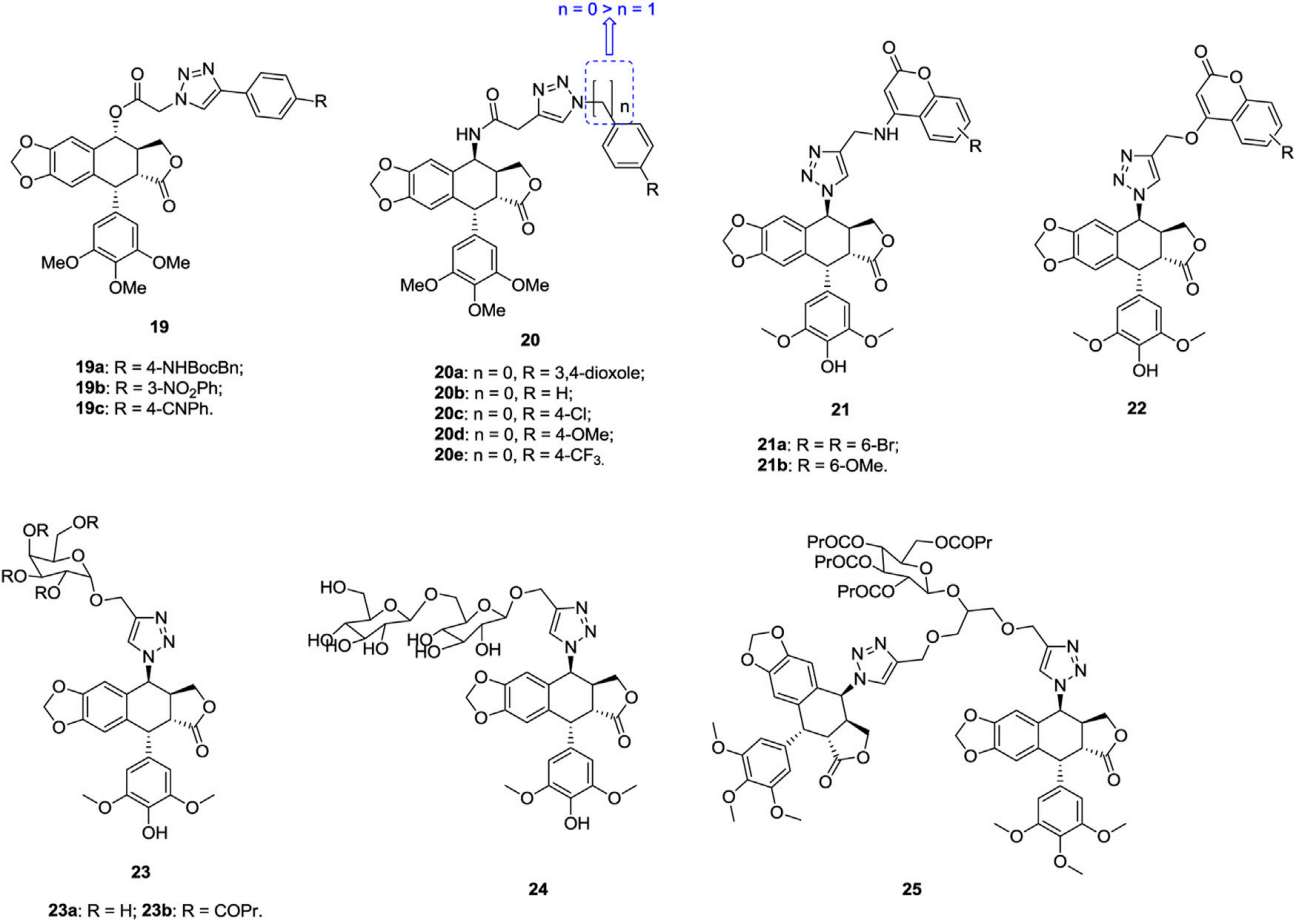
Chemical structures of 1,2,3-triazole–containing (epi)podophyllotoxin derivatives 19–25.

1,2,3-Triazole–containing podophyllotoxin derivatives **19** (Figure 5, IC_50_: 21.1–118.8 nM, MTT assay) are highly potent against A549 cells, and compounds **19a-c** (IC_50_: 21.1–29.4 nM) are comparable to podophyllotoxin (IC_50_: 27.1 nM) (Hou et al., 2019). Mechanistic investigations show that hybrid **19a** could exert antiproliferative activity through acting on microtubules, causing cell cycle arrest at the G2/M phase, and inducing apoptosis cancer cells.

1,2,3-Triazole–containing epipodophyllotoxin derivatives **20** (IC_50_: 0.97–34.46 μm, MTT assay) exhibit considerable activity against A549 cells, and an SAR study implies that introduction of a carbon spacer between 1,2,3-triazole and phenyl ring reduces the activity (Reddy et al., 2018). Compounds **20a-e** (IC_50_: 0.97–1.96 μm) possess higher activity than the references podophyllotoxin (IC_50_: 4.60 μm) and etoposide (IC_50_: 1.97 μm), and the cytotoxicity (IC_50_: 53.86–89.04 μm) toward NIH/3T3 cells is much lower. A mechanistic study demonstrates that these hybrids could inhibit topoisomerase II, arrest G2/M phase of cell cycle, and induce apoptosis.

1,2,3-Triazole–tethered podophyllotoxin-coumarin hybrids **21** (IC_50_: 8.6–34.8 μm, MTT assay) show higher antiproliferative activity than their analogues **22** (IC_50_: 19.6->100 μm) against A549 cells, revealing that the amino linker to couple 1,2,3-triazole with coumarin moieties is more favorable than ether linker (Hao et al., 2019). In addition, substituents into the C-6 position of the coumarin moiety and the electron-withdrawing group are beneficial for the activity. In particular, hybrids **21a,b** (IC_50_: 8.6 and 17.5 μm) are found much more potent than the reference etoposide (IC_50_: 25.6 μm) against A549 cells, and hybrid **21b** could disrupt microtubules and induce cell cycle arrest at the G1 phase by regulating P21 and cyclin D1.

1,2,3-Triazole–tethered epipodophyllotoxin-galactose hybrid **23a** possesses potent broad-spectrum antiproliferative activity, and the activity against A549 cells (IC_50_: 4.07 μm, MTT assay) is 2-fold higher than that of cisplatin (IC_50_: 9.24 μm) and etoposide (IC_50_: 11.92 μm) (Zi et al., 2015). An SAR study reveals that butyrylation of the galactose moiety generally benefits for the activity, as evidenced by that hybrid **23b** (IC_50_: 1.52 μm) is six times superior to cisplatin (IC_50_: 9.24 μm) and etoposide (IC_50_: 11.92 μm) against A549 cells (Zi et al., 2015).

The majority of 1,2,3-triazole–tethered epipodophyllotoxin-*bis*-glucose hybrids (IC_50_: > 40 μm, MTT assay) are devoid of activity against A549 cells, but hybrid **24** (IC_50_: 3.84 μm) demonstrates higher activity than the references cisplatin (IC_50_: 6.15 μm) and etoposide (IC_50_: 11.92 μm) (Zi et al., 2017). Similar results are also observed for *bis*-1,2,3-triazole–tethered *bis*-epipodophyllotoxin-glucose hybrids (IC_50_: > 40 μm, MTT assay). Only hybrid **25** (IC_50_: 0.89 μm) displays excellent inhibitory activity against A549 cells, and the activity is 6.9- and 13.3-folds superior to those of cisplatin (IC_50_: 6.15 μm) and etoposide (IC_50_: 11.92 μm) (Zi et al., 2018). This hybrid (CC_50_: 15.38 μm) also shows relatively low cytotoxicity against normal BEAS-2B cells, and the SI is 17.2. Accordingly, hybrid **25** could serve as a lead compound for further investigations.

## 1,2,3-Triazole–Containing Quinoline/Quinolone Derivatives

Quinoline and quinolone derivatives are potential inhibitors of hepatocyte growth factor receptor, proto-oncogene receptor tyrosine kinase/KIT, platelet-derived growth factor receptor-*β*/PDGFR-*β* and VEGFR2, and some quinoline-/quinolone-based agents, such as anlotinib and lenvatinib, have already been approved for lung cancer therapy (Jain et al., 2019; Li and Zhu, 2021). Hence, combination of 1,2,3-triazole with quinoline/quinolone may provide valuable therapeutic drug candidates against lung cancer.

**FIGURE 6:**
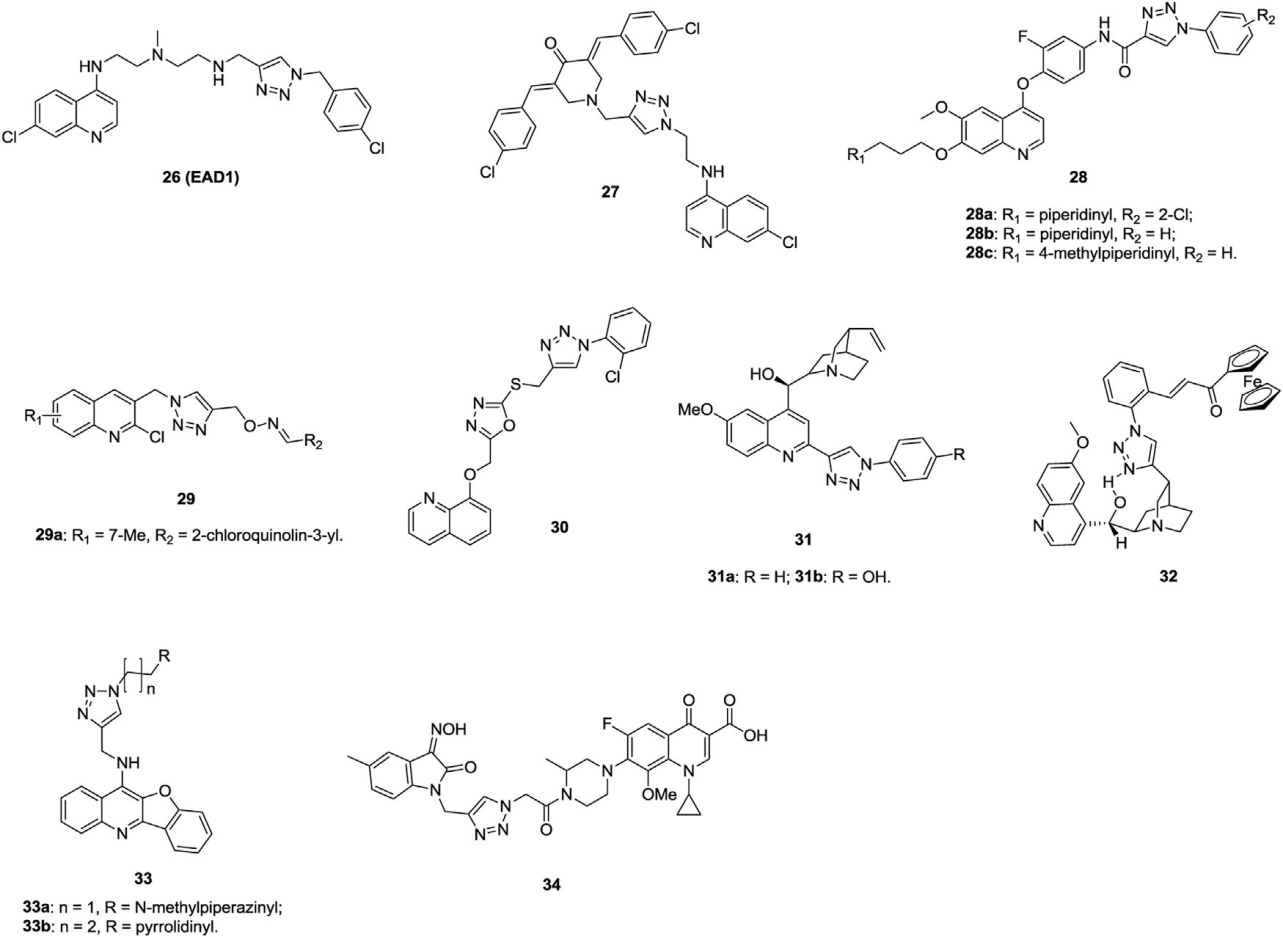
Chemical structures of 1,2,3-triazole–containing quinoline/quinolone derivatives 26–34.

The majority of 1,2,3-triazole–containing quinoline derivatives (IC_50_: 7.6–164 μm, SAR assay) are active against H460 and HCC827 human lung cancer cell lines, and compound **26** (**EAD1**, Figure 6, IC_50_: 11 and 7.6 μm) is more potent than chloroquinoline (IC_50_: 52 and 76 μm) (Nordstrøm et al., 2015). A mechanistic study indicates that compound **26** exerts antiproliferative activity by inhibiting autophagy and inducing apoptosis. Compound **27** (GI_50_: 0.189–2.17 μm, SRB assay) demonstrates promising activity against a series of cancer cell lines, including eight NSCLC (GI_50_: 0.25–1.91 μm), and a mechanistic study reveals that this hybrid could induce apoptosis (Kandi et al., 2015). Compounds **28** (IC_50_: 0.07–3.50 μm, MTT assay) display potential activity against A549 cells, and an SAR study elucidates that the piperidinyl group at the R_1_ position enhances the activity (Liu et al., 2016). Among them, compounds **28a-c** (IC_50_: 0.07–0.14 μm) are not inferior to foretinib (IC_50_: 0.11 μm). In particular, the most active compound **28a** with an IC_50_ value of 2.27 nM against c-Met is identified as a multitargeted receptor tyrosine kinase inhibitor. Thus, this compound is worthy of further investigation. Some 1,2,3-triazole–containing 2-chloroquinoline derivatives, such as compound **29** (IC_50_: 9.8 μm against A549 cells, MTT assay; 9.7 μm for doxorubicin), also display certain antiproliferative activity against lung cancer cells, but most of them are less potent than the references (Praveena et al., 2016; Dasari et al., 2019). Further modifications implies that incorporation of azole between 1,2,3-triazole and quinoline moieties is also allowed, and 1,2,3-triazole–tethered 1,3,4-oxadiazole-quinoline compound **30** (IC_50_: 5.6 μm, MTT assay) is comparable to doxorubicin (IC_50_: 1.83 μm) against A549 cells (Rachakonda et al., 2017; Shamsi et al., 2019).

The antiproliferative activity of 1,2,3-triazole–containing quinine derivatives **31a,b** (IC_50_: 3.3 and 4.7 μm, MTT assay) is comparable to that of cisplatin (IC_50_: 3.5 μm) against A549 cells (Boratynski et al., 2018), while compound **32** (IC_50_: 2.34 and 2.13 μm, MTT assay) shows promising activity against drug-sensitive NCI-H460 and MDR NCI-H460/R; probably due to that this compound could increase ROS production and induce mitochondrial damage in MDR cancer cells, demonstrating its potential against MDR lung carcinoma (Podolski-Renic et al., 2017).

Aside from the 1,2,3-triazole–containing quinoline derivatives mentioned above, some other derivtives are also endowed with certain activity against lung cancer cells (Theeramunkong et al., 2016; Zeng et al., 2017; Qiao et al., 2019; Rono et al., 2019). 1,2,3-Triazole–containing benzofuroquinoline derivatives **33a,b** (IC_50_: 0.53 and 0.26 μm, MTT assay) exhibit excellent activity against A549 cells and low cytotoxicity (IC_50_: 25.24 and 16.05 μm) toward mouse mesangial cells (Zeng et al., 2017). A mechanistic study reveals that compound **33b** could exert antiproliferative activity by inducing G0/G1 phase arrest and down-regulating *c*-*myc* gene transcription.

1,2,3-Triazole–tethered ciprofloxacin/gatifloxacin/moxifloxacin-isatin hybrids are also active against A549 cells, and hybrid **34** (IC_50_: 44.2 μm, CCK-8 assay) is more potent than vorinostat (IC_50_: 76.3 μm), revealing that these types of hybrids could be taken for further investigations (Chen et al., 2019; Jiang and Zhang, 2019; Yang et al., 2020).

## 1,2,3-Triazole–Containing Pyridine Derivatives

Pyridines are potential inhibitors of CDK, EGFR, PI3K, and RGGT, and some pyridine-based agents, such as masitinib, have already been applied in clinical practice or under clinical trials for the treatment of cancers (Goel et al., 2016; Prachayasittikul et al., 2017). Thus, combination of 1,2,3-triazole with pyridine may provide opportunities for the development of novel anticancer agents.

**FIGURE 7:**
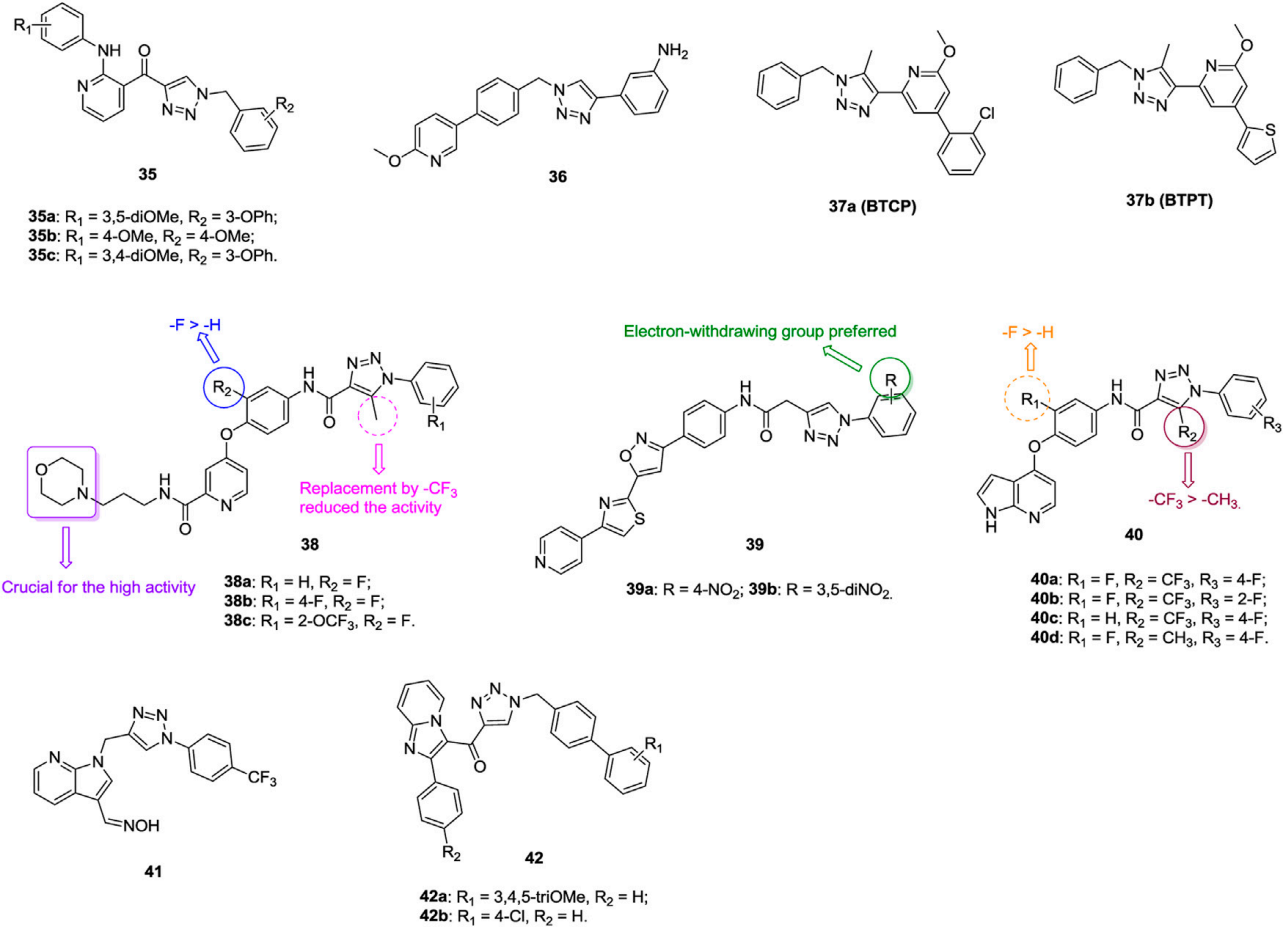
Chemical structures of 1,2,3-triazole–containing pyridine derivatives 35–42.

An antiproliferative SAR of 1,2,3-triazole–containing pyridine derivatives **35** (Figure 7, IC_50_: 1.023–23.61 μm, MTT assay) against A549 cells indicates that the methoxy group on the 2-arylpyridine moiety (R_1_ position) and the 3-phenoxy group on the benzyl group are favorable for the activity (Kamal et al., 2015; Prasad et al., 2019). The most active compounds **35a**-**c** (IC_50_: 1.023–1.148 μm) exhibit higher activity than the reference E7010 (IC_50_: 1.622 μm), and mechanistic studies reveals that these compounds could inhibit the microtubule assembly, arrest the cell cycle at the G2/M phase, and induce cell death by apoptosis. **BD7** (**36**, IC_50_: 0.07–0.49 μm, MTT assay) shows potent antiproliferative activity against various cancer cell lines, and its activity is identical to that of sorafenib (IC_50_: 0.08–0.30 μm) (Pan et al., 2019). Biological results indicate that **BD7** displays simultaneous inhibition of VEGFR-2, Tie-2, and EphB4. Thus, this compound could be considered as a promising template for further optimization of 1,2,3-trizole–incorporated derivatives as VEGFR-2/Tie-2/EphB4 inhibitors. **BTCP** (**37a**, IC_50_: 42.6 μm, MTT assay) is not inferior to doxorubicin (IC_50_: 38.2 μm) against A549 cells, and **BTPT** (**37b**, IC_50_: 0.68 μm, MTT assay) with the thiophene moiety at the *para* position of the pyridine moiety holds enhanced activity against A549 cells (Murugavel et al., 2019; Murugavel et al., 2019).

An antiproliferative SAR study of 1,2,3-triazole–containing pyridine derivatives **38** against A549 cells demonstrates that the methyl group on the 1,2,3-triazole motif and the fluoro on the phenyl ring are advantageous to the activity (Xiong et al., 2020). The morpholino group is essential for high activity, while replacement by pyrrolidinyl, thienyl, and alkyl groups leads to great loss of activity. In particular, compounds **38a-c** (IC_50_: 3.22–6.43 μm, MTT assay) are more potent than golvatoinib (IC_50_: 8.14 μm), and the most active compound **38b** could block cells in the G0/G1 phase. 1,2,3-Triazole–containing isoxazole–thiazole–pyridine hybrids **39** (IC_50_: 0.01–10.22 μm, MTT assay) has promising activity against A549 cells, and an SAR study implies that electron-withdrawing groups, especially the nitro group on the phenyl ring, could greatly increase the activity (Yakantham et al., 2019). The most active hybrid **39b** (IC_50_: 0.01 μm) is 308-fold more potent than etoposide (IC_50_: 3.08 μm) against A549 cells, so it could serve as a lead compound for further development of novel anti–lung cancer candidates.

1,2,3-Triazole–containing pyrrolo(2,3-b)pyridines **40** (IC_50_: 0.082–2.83 μm, MTT assay) exhibit excellent activity against A549 cells, and an SAR study demonstrates that the fluoro at the R_1_ position and the trifluoromethyl group at the R_2_ position are beneficial for the activity (Tang et al., 2016; Wang et al., 2018). The activity of representative compounds **40a-d** (IC_50_: 0.082–0.19 μm, MTT assay) is higher than that of foretinib (IC_50_: 0.49 μm), and compound **40a** (IC_50_: 1.68–4.57 nM) shows excellent inhibitory activity against c-Met, Flt-3, and PDGFR-*β*. Further study disclosed that incorporation of oxime into the pyrrolo(2,3-b)pyridine moiety (IC_50_: 0.12–3.84 μm, MTT assay) is permitted, and the most active compound **41** (IC_50_: 0.12 μm) could intercalate into calf thymus DNA efficiently to form a **41**-DNA complex that might block DNA replication to exert its antiproliferative activity (Narva et al., 2016).

1,2,3-Triazole–containing imidazopyridines **42** (IC_50_: 0.51–47.92 μm, MTT assay) show considerable activity against A549 cells, and an SAR study illustrated that a substitution at the R_2_ position, regardless if the substituent is an electron-donating or electron-withdrawing group, could reduce the activity as compared with hydrogen (Sayeed et al., 2018). In particular, compounds **42a,b** (IC_50_: 0.51 and 0.63 μm) are more potent than nocodazole (IC_50_: 1.47 μm) against A549 cells. Flow cytometry reveals that these compounds result in A549 cell cycle arrest at the G2/M phase, and further studies indicates that these compounds could inhibit tubulin and induce cell death by apoptosis.

## 1,2,3-Triazole–Containing Pyrimidine/Quinazolinone/Nucleoside Derivatives

Pyrimidines, quinazolinones, and nucleosides could inhibit various MDR proteins, such as P-gp and MDR-associated protein-1, and several pyrimidine-/quinazolinone-/nucleoside-based agents, such as azacitidine and gemcitabine, demonstrate high efficacy for a broad spectrum of cancers (Robak, 2011; Patil, 2018; He et al., 2020). Therefore, 1,2,3-triazole–containing pyrimidine/quinazolinone/nucleoside derivatives may have the potency to overcome MDR, and they may represent promising leads for the development of novel anticancer agents.

**FIGURE 8:**
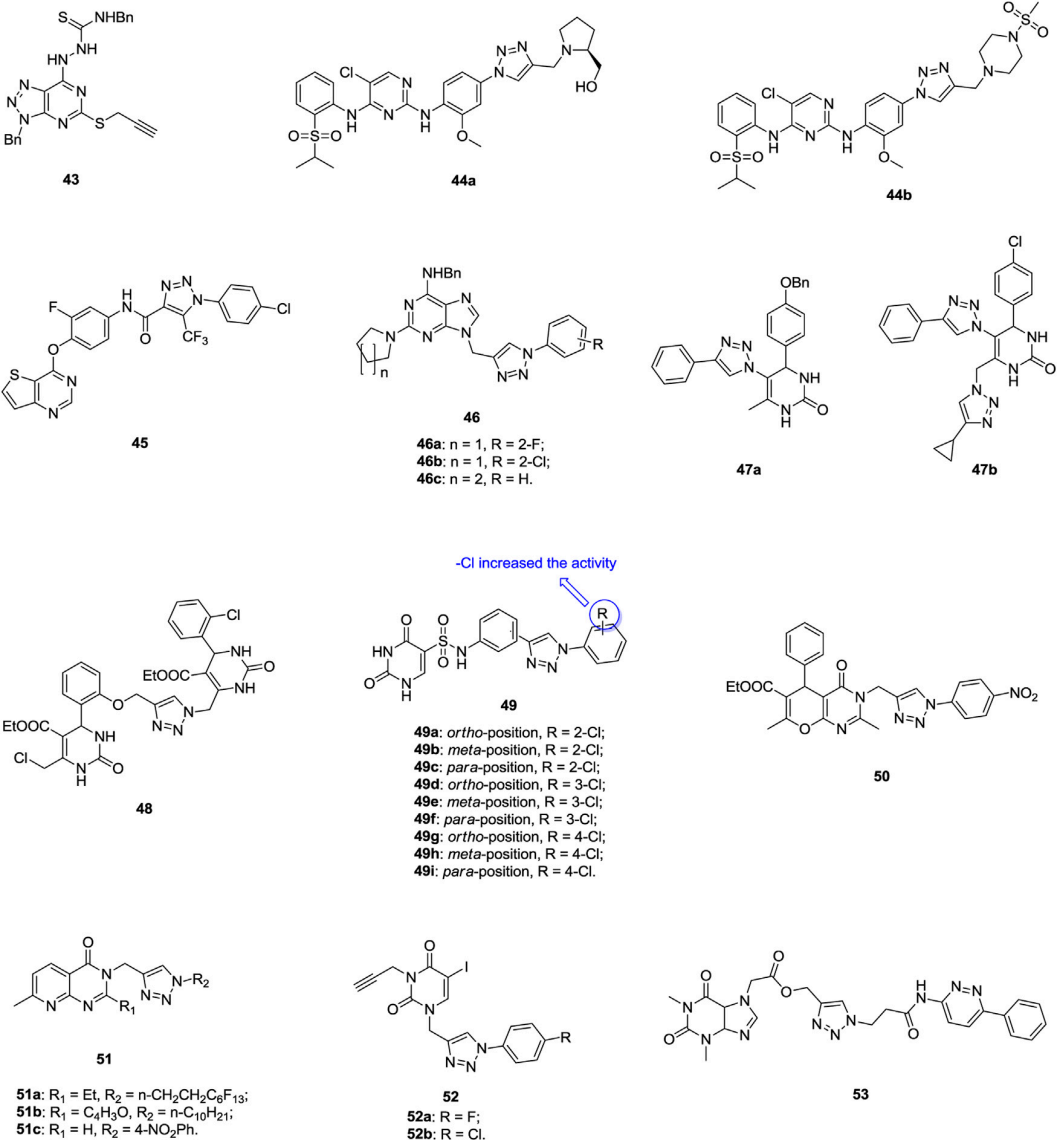
Chemical structures of 1,2,3-triazole–containing pyrimidine derivatives 43–53.

1,2,3-Triazolo(4,5-d)pyrimidine **43** (Figure 8, IC_50_: 2.37 μm, MTT assay) is 3.3 folds more potent than 5-flurouracil (IC_50_: 7.86 μm) against NCI-H1650 cells, and mechanistic studies disclose that this compound can inhibit the migration of cancer cells and induce apoptosis (Geng et al., 2018). 1,2,3-Triazole–containing pyrimidine derivatives **44a,b** (IC_50_: 0.29–0.91 μm, MTT assay) hold potential activity against A549 and the NSCLC cell line H2228 expressing EML4-ALK, and both of them display superior activity to crizotinib (IC_50_: > 1 μm) and ceritinib (IC_50_: > 1 μm) against A549 cells (Wang et al., 2018). Further study indicates that these compounds could induce cell apoptosis and inhibit cellular ALK and ROS1 activities. Accordingly, these compounds could act as promising ALK and ROS1 dual inhibitors to overcome crizotinib-resistant mutants. 1,2,3-Triazole-pyrrolopyrimidine/imidazo(2,3-d)pyrimidine/thieno(3,2-d)pyrimidines also possess certain activity against lung cancer cell lines, and among them, compound **45** (IC_50_: 0.9 μm, MTT assay) is 5.2 times more potent than foretinib (IC_50_: 4.7 μm) against A549 cells (Lee et al., 2016; Bistrovic et al., 2018; Wang et al., 2018). A mechanistic investigation reveals that compound **45** could induce apoptosis in a concentration-dependent manner (Wang et al., 2018). The majority of 1,2,3-triazole–containing purine derivatives **46** (IC_50_: 0.03–45.2 μm, SRB assay) display inhibitory activity against A549 cells, and three compounds **46a-c** (IC_50_: 0.03–0.14 μm) are 35–163.3 folds more potent than 5-fluorouracil (IC_50_: 4.9 μm), revealing the potential of these compounds as novel anti–lung cancer agents (Khazir et al., 2020).

1,2,3-Triazole–containing dihydropyrimidinone **47a** (GI_50_: 14 and 18 μm, SRB assay) and its derivative **47b** (GI_50_: 15 and 17 μm) show considerable activity against SW1573 and A549 lung cancer cell lines (Caeeeiro et al., 2020), but incorporation of the second dihydropyrimidinone fragment does not positively affect the activity; as evidenced by that compound **48** (IC_50_: 20 μm, MTT assay) shows moderate activity against A549 cells (Shamsiya and Damodaran, 2019). 1,2,3-Triazole–containing dihydropyrimidinones **49a-i** (IC_50_: 1.18–2.81 μm, MTT assay) are more potent than pemetrexed (IC_50_: 3.29 μm) against A549 cells, and an SAR study illustrates that the chloro on the phenyl rings enhance the activity (Lu et al., 2019). Flow cytometric analysis shows that compound **49g** could inhibit the proliferation of A549 cells by arresting the cell cycle in the G1/S phase and inducing cell apoptosis. The 1,2,3-triazole–containing 3*H*-pyrano(2,3-d)pyrimidinone-6-carboxylate **50** (IC_50_: 0.69 μm, MTT assay) shows slightly lower potency than the reference doxorubicin (IC_50_: 0.14 μm) against A549 cells, demonstrating its potential as a lead compound for further development of new therapeutic drug candidates (Boda et al., 2018). 1,2,3-Triazole–containing pyrido(2,3-d)pyrimidinones **51** (GI_50_: 0.03–5.33 μm, SRB assay) are active against A549 cells, and the most active compound **51a** (GI_50_: 0.03 μm) is more potent than the reference nocodazole (GI_50_: 0.08 μm) (Kumar et al., 2016). Similar activity of compounds **52a,b** (IC_50_: 3.06 and 4.69 μm, MTT assay) to 5-fluorouracil (IC_50_: 2.80 μm) is also observed, but both the compounds **52a,b** (IC_50_: 2.22 and 0.04 μm) display high cytotoxicity toward normal NIH 3T3 cells (Gregoric et al., 2017). Compound **53** (IC_50_: 1.34 μm, MTT assay) also exhibits considerable activity against A549 cells, but it is less potent than combretastatin-A4 (IC_50_: 0.11 μm) (Ruddarraju et al., 2017).

**FIGURE 9:**
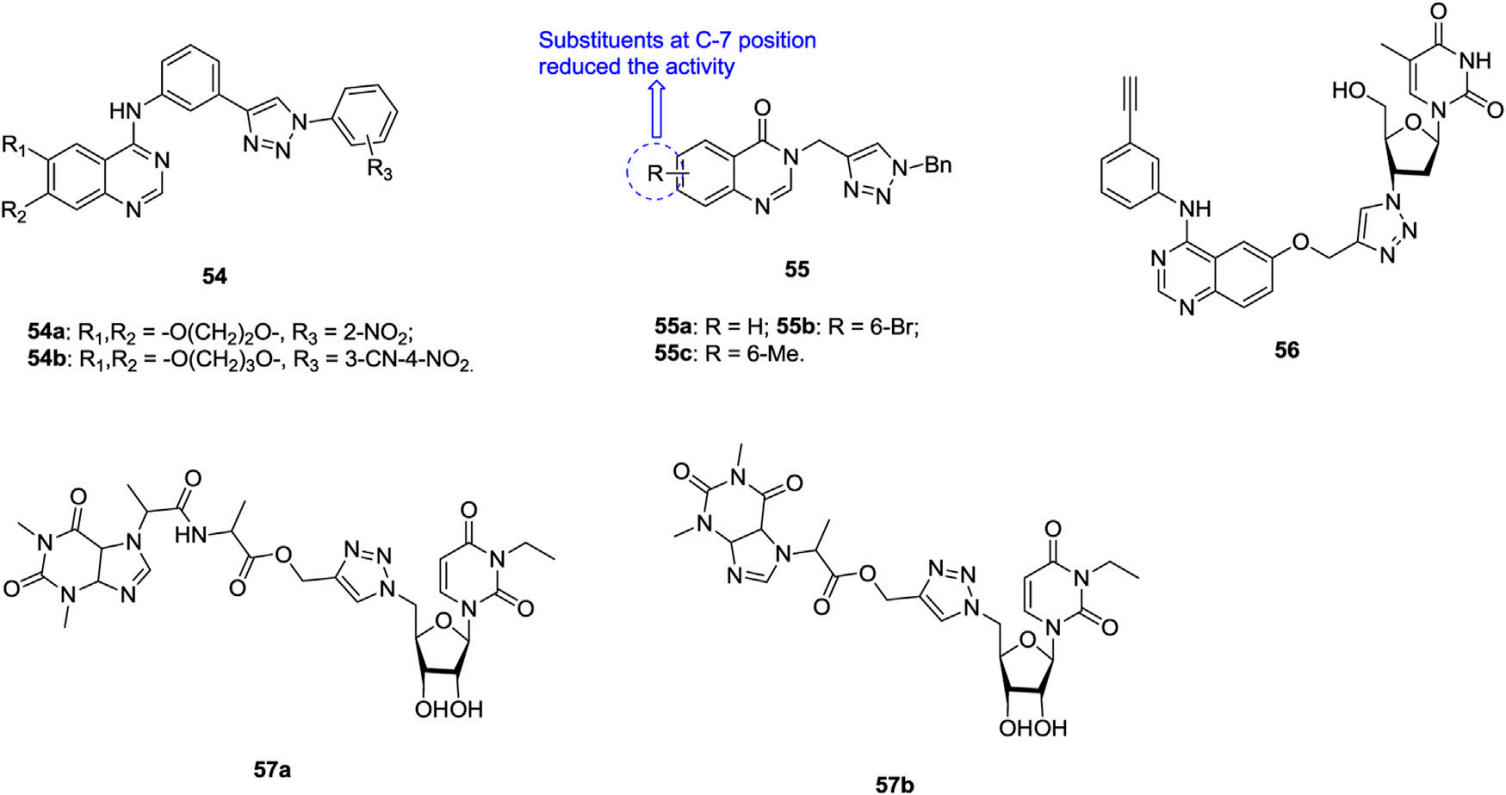
Chemical structures of 1,2,3-triazole–containing quinazoline/nucleoside derivatives 54–57.

Most of 1,2,3-triazole–containing quinazoline derivatives **54** (Figure 9, IC_50_: 1.03–9.16 μm, MTT assay) possess potential activity against SK-Lu-1 NSCLC, and the activity is superior to that of erlotinib (IC_50_: 99.76 μm) (Le-Nhat-Thuy et al., 2018). An SAR study implies that the introduction of a nitro group into the *ortho* position of the phenyl ring at the N-1 position of the 1,2,3-triazole moiety could boost the activity to some extent. The representative compounds **54a,b** (IC_50_: 1.03 and 1.81 μm) possess comparable activity to ellipticine (IC_50_: 1.38 μm), and molecular docking studies indicates that these compounds could exert antiproliferative activity through targeting EGFR.

Some 1,2,3-triazole–containing quinazolin-4(3*H*)-one derivatives also demonstrate considerable activity against lung cancer cell lines, and an antiproliferative SAR study of compounds **55** (IC_50_: 2.01–12.34 μm, MTT assay) against NCI-H23 lung cancer cells elucidates that introduction of substituents into the quinazolin-4(3*H*)-one motif, especially at the C-7 position, is unfavorable to the activity (Safavi et al., 2018; Vasu et al., 2018; Lan et al., 2020). No inferior activity is found for compounds **55a-c** (IC_50_: 2.01–2.69 μm) compared with doxorubicin (IC_50_: 1.29 μm) and SAHA (IC_50_: 1.44 μm), and compounds **55a-c** are around six folds more potent than 5-fluorouracil (IC_50_: 13.45 μm). The most active compound **55a** could induce early apoptosis and arrest at the G2/M phase. Thus, it could serve as a new lead for the design and development of potent anticancer agents.

The majority of 1,2,3-triazole–tethered azidothymidine–quinazoline hybrids (IC_50_: > 200 μm, MTT assay) are devoid of activity against SK-Lu-1 NSCLC, whereas the activity of hybrid **56** (IC_50_: 9.06 μm, MTT assay) is at the same level as that of ellipticine (IC_50_: 1.30 μm), and 3.4 times higher than that of erlotinib hydrochloride (IC_50_: 31.15 μm) (Giang et al., 2018). The 1,2,3-triazole–tethered nucleoside-theophylline hybrids **57a,b** (IC_50_: 1.56 and 2.89 μm, MTT assay) also display certain activity against A549 cells, but the activity is considerably inferior to that of combretastatin-A4 (IC_50_: 0.11 μm) (Ruddarraju et al., 2016; Trznadel et al., 2019).

## 1,2,3-Triazole–Containing Steroid Derivatives

Steroids that are ubiquitous in natural resources are involved in several physiological functions and exhibit low cytotoxicity, high bioavailability, and excellent efficiency against diverse cancers, including lung cancers (Tantawy et al., 2017; Xiao et al., 2020). Thus, combination of 1,2,3-triazole with steroid is an attractive strategy to develop novel anticancer drug candidates.

**FIGURE 10:**
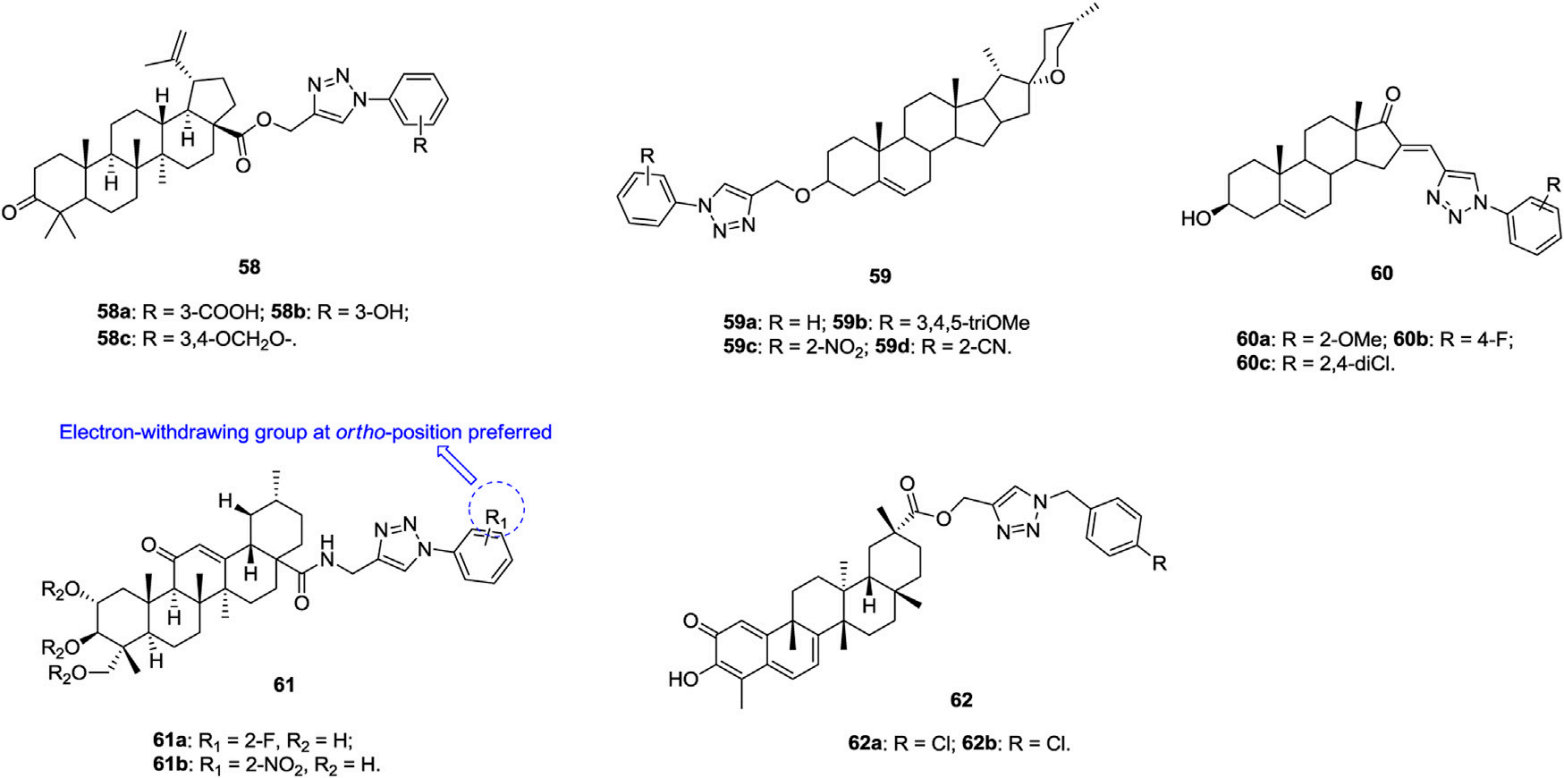
Chemical structures of 1,2,3-triazole–containing steroid derivatives 58–62.

1,2,3-Triazole–containing betulinic acid derivatives **58** (Figure 10, IC_50_: 3.7–9.0 μm, MTT assay) show promising cytotoxic potential against A549 cells, and the activity is higher than that of the parent betulinic acid (IC_50_: 23.0 μm) (Khan et al., 2016; Dangroo et al., 2017; Grishko et al., 2017). An SAR study suggested that hydrogen bond donors, such as hydroxyl and carboxylic acid, at the *meta* position of the phenyl ring on the N-1 position of the 1,2,3-triazole moiety are beneficial for their activity. Further studies demonstrated that compound **58a** could arrest the G1 phase of the cell cycle and induce apoptosis, which confers its anti-invasive and anti-metastatic behavior toward cancer cells. An antiproliferative SAR study of 1,2,3-triazole–containing diosgenin derivatives **59** (IC_50_: 5.54–31.00 μm, MTT assay) against A549 cells indicates that introduction of electron-donating or electron-withdrawing groups into the phenyl ring is detrimental to the activity (Masood-Ur-Rahman et al., 2017). Among them, four compounds **59a-d** (IC_50_: 5.54–7.23 μm) are comparable to BEZ-235 (IC_50_: 6.52 μm) but more potent than diosgenin (IC_50_: 13.3 μm). Most 1,2,3-triazole–containing dehydroepiandrosterone derivatives **60** (IC_50_: 7.6–75.2 μm, MTT assay) are active against A549 cells, and these compounds exert antiproliferative effects by arresting cells in the G2 phase of the cell cycle and inducing apoptosis (Huang et al., 2018).

1,2,3-Triazole–containing asiatic acid derivatives **61** (IC_50_: 2.67–39.87 μm, MTT assay) show promising activity against A549, NCI-H460, and NCI-H460/DOX lung cancer cell lines, and an SAR study reveals that the electron-withdrawing group at the *ortho* position of the phenyl ring is favorable to the activity (Huang et al., 2019). The representative compound **61a** (IC_50_: 2.67–4.84 μm) is the most active against the three tested lung cancer cell lines, and the RI value is 1.08, suggesting its potential to fight against drug-resistant lung cancer. Moreover, compound **61a** (IC_50_: > 50 μm) is nontoxic toward normal HL-7702 cells, and the SI values are > 10.3. Mechanistic studies illustrate that compound **61a** is a potential NF-κB inhibitor and could induce apoptosis and suppress cell migration. Accordingly, rational design of 1,2,3-triazole–containing asiatic acid derivatives may offer a new class of NF-κB inhibitors with the ability to suppress cancer cell migration and induce apoptosis.

Apart from the 1,2,3-triazole–containing steroid derivatives discussed above, some other derivatives also possess certain activity against lung cancer cell lines (Zhu et al., 2015; Zhang et al., 2018; Popov et al., 2020; Treyakova et al., 2020). 1,2,3-Triazole–containing celastrol derivatives **62a,b** (IC_50_: 0.97–54.94 μm, MTT assay) show broad-spectrum antiproliferative activity, and compound **62a** (IC_50_: 3.53 μm) is comparable to celastrol (IC_50_: 3.02 μm) against A549 cells (Zhang et al., 2018). An SAR study demonstrates that the 1,2,3-triazole moiety is not essential for the activity and that the removal of this moiety exerts minimal influence on the activity.

## Miscellaneous 1,2,3-Triazole–Containing Compounds

**FIGURE 11:**
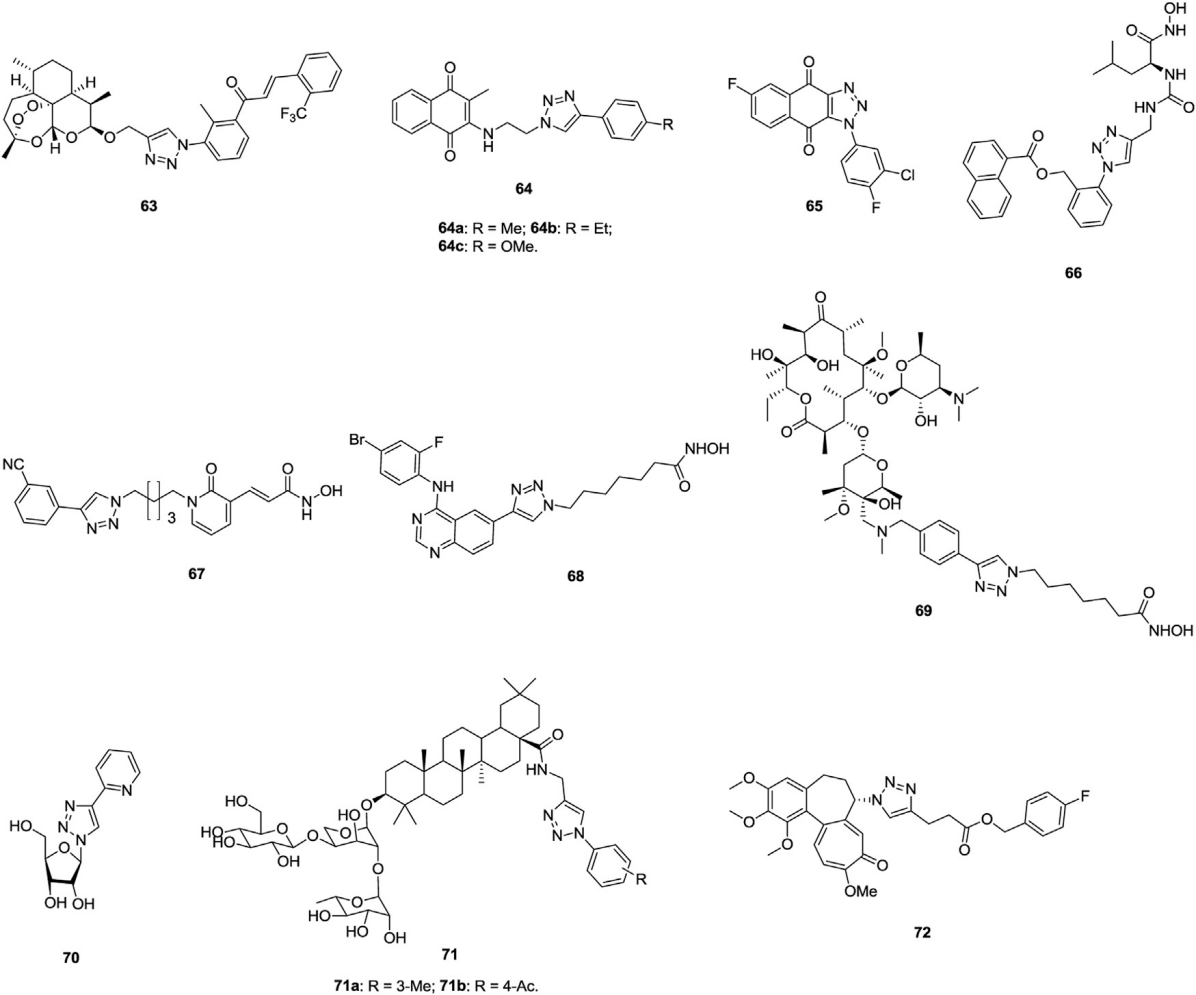
Chemical structures of 1,2,3-triazole–containing compounds 63–72.

1,2,3-Triazole–tethered dihydroartemisinin–chalcone hybrid **63** (Figure 11, IC_50_: 7.16 μm, MTT assay) display comparable activity to doxorubicin (IC_50_: 6.36 μm), but it is more potent than dihydroartemisinin (IC_50_: 43.43 μm) against A549 cells, and this hybrid (IC_50_: > 100 μm) is nontoxic toward human erythrocyte and normal kidney cells (HEK-293) (Kapkoti et al., 2018). An SAR study implies that incorporation of the second dihydroartemisinin moiety could boost the activity to some extent. Mechanistic studies reveal that hybrid **63** significantly induces the ROS formation in A549 cells and causes cell cycle arrest at the G2/M phase as well as apoptosis in cancer cells.

1,2,3-Triazole–containing naphthoquinones **64** (IC_50_: 8.33–84.14 μm, MTT assay) show considerable activity against A549 cells, and an SAR study demonstrated that the alkyl or alkyloxy group at the *para* position of the phenyl ring has advantages to the activity (Prasad et al., 2018). Among them, compound **64b** (IC_50_: 9.19 μm) is slightly more active than tamoxifen (IC_50_: 10.87 μm), and it could arrest the cell cycle at the G0/G1 phase and induce apoptotic cell death. Accordingly, compound **64b** could be taken as a lead molecule for further development of potent anti–lung cancer therapeutic agents. In LL2 (Lewis lung cancer, bearing a high expression level of IDO1) and Hepa1-6–xenografted mouse models, compound **65** (30 mg/kg, intraperitoneally) demonstrates moderate *in vivo* anticancer efficacy with TGI rates of 62.5% and 80%, and the potency is higher than that of epacdostat (TGI: 50.0%) (Pan et al., 2020). Moreover, *in vivo* experiments reveal no obvious body weight change, implying its excellent safety profile. Therefore, compound **65** could serve as a preclinical candidate for further evaluations.

1,2,3-Triazole–containing hydroxamic acid **66** (IC_50_: 57.5 μm, MTT assay) not only displays comparable activity to 5-fluorouracil (IC_50_: 37.9 μm) against A549 cells but also exhibits synergistic effect with 5-fluorouracil (IC_50_: 16.6 μm) (Cao et al., 2019). Further studies showed that compound **66** (IC_50_: 32 nM) is a potential aminopeptidase N inhibitor, and it is nontoxic toward normal HUVECs (IC_50_: > 2000 μm). In addition, in the H22-xenografted mouse model, compound **66** alone or in combination with 5-fluorouracil could effectively inhibit tumor growth without causing loss of body weight. 1,2,3-Triazole–tethered dihydropyridin-2-one-hydroxamic acid hybrid **67** (IC_50_: 35 and 49 nM) is a potential HDAC1 and HDAC6 inhibitor, and it (IC_50_: 8.8 μm, MTT assay) is as potent as SAHA (IC_50_: 8.9 μm) against A549 cells (Li and Han, 2016). Moreover, hybrid **67** (IC_50_: > 50 μm) is nontoxic toward normal RWPE-1 and VERO cells, and SI > 5.6. Compound **68** reveals outstanding potency (IC_50_: 1.1–10.3 nM) against EGFR, HDAC1, and HDAC6 and promising activity (IC_50_: 0.71 and 7.85 μm, MTT assay) against A549 and NCI-H1975 lung cancer cell lines, and its antiproliferative activity is also superior to that of vorinostat (IC_50_: 2.67 and 23.76 μm) (Ding et al., 2017). Thus, this compound could act as a potential candidate for clinical applications. 1,2,3-Triazole–tethered clarithromycin-hydroxamic acid hybrid **69** (IC_50_: 23.9 and 2.85 nM) demonstrates excellent inhibitory activity against HDAC1 and HDAC6, and the activity of this hybrid (IC_50_: 0.99 and 0.69 μm, MTT assay) is around five-fold higher than that of SAHA (IC_50_: 5.00 and 3.27 μm) against A549 cells (Tapadar et al., 2015).

Some 1,2,3-triazole–containing sugar derivatives also possess certain antiproliferative activity against lung cancer cell lines (Petrova et al., 2015; Nagarsenkar et al., 2016; Li et al., 2018; Jakukowski et al., 2020; Tsai et al., 2020; Zi et al., 2020), and 1,2,3-triazole–tethered β-**D**-ribofuranose-pyridine hybrid 70 (IC_50_: 17.7 μm, MTT assay) is 3.4-fold more potent than cisplatin (IC_50_: 17.7 μm) against A549 cells (Jakukowski et al., 2020), whereas hybrids **71** (IC_50_: 0.18–54.89 μm, MTT assay) are more active than 5-fluorouracil (IC_50_: 69.07 μm) (Li et al., 2018). An SAR study suggests that the electron-donating group benefits the activity compared with the electron-withdrawing group on the phenyl ring, and replacement of the phenyl ring by the pyridinyl ring results in significant loss of activity. In particular, hybrids **71a,b** (IC_50_: 0.18 and 0.28 μm) are highly potent against A549 cells, and mechanistic investigations demonstrate that these hybrids could inhibit the proliferation by inducing apoptosis and arresting the cell cycle at the G1 and S phases.

1,2,3-Triazole–containing colchicine derivative **72** (IC_50_: 3.5 nM, MTT assay) shows remarkable cytotoxic efficacy against A549 cells, and the activity is 3.7-fold higher than that of paclitaxel (IC_50_: 13.2 nM), revealing the potential of 1,2,3-triazole–containing colchicine derivatives as novel anti–lung cancer candidates (Thomopoulou et al., 2016).

**FIGURE 12:**
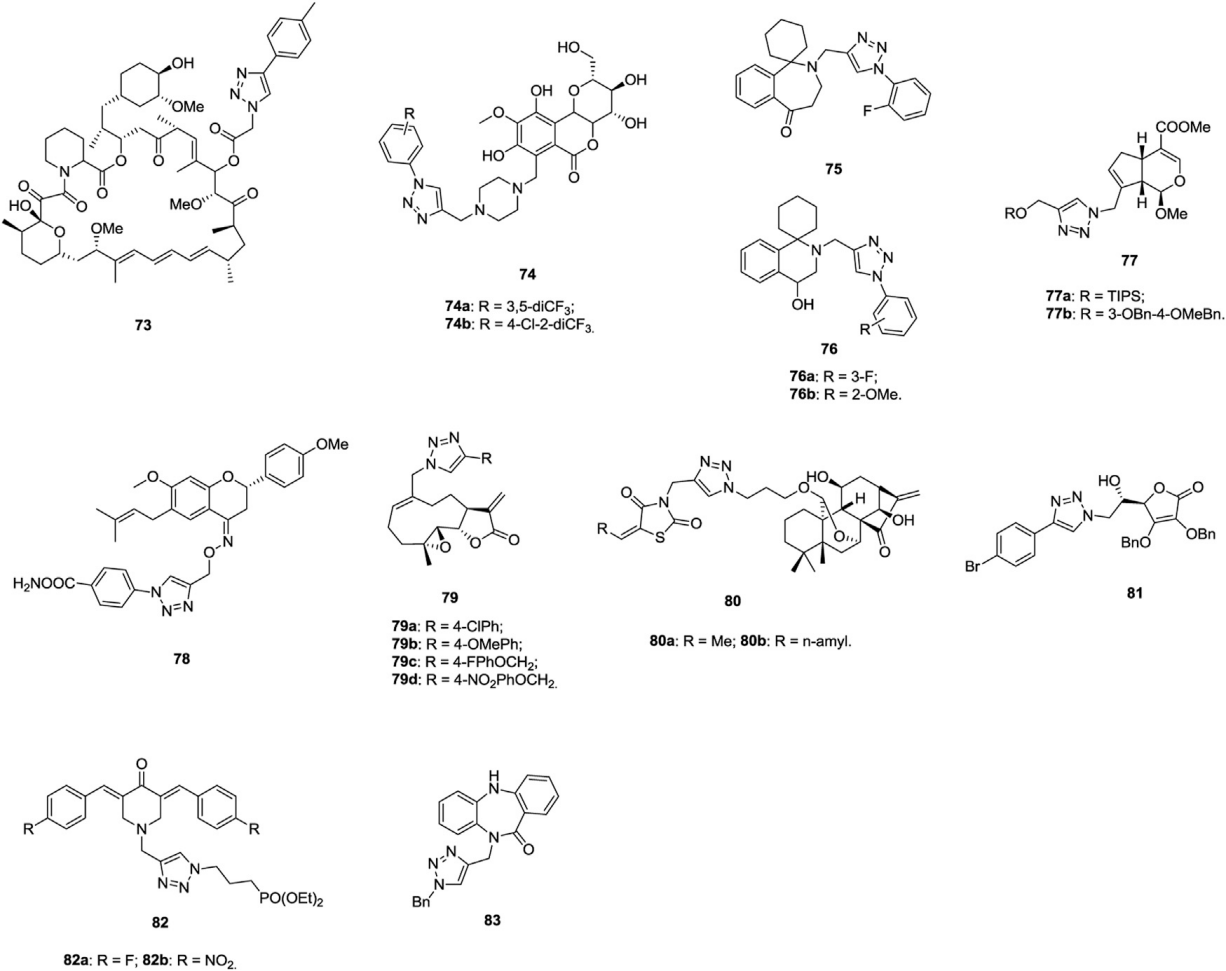
Chemical structures of 1,2,3-triazole–containing compounds 73–83.

1,2,3-Triazole–containing rapamycin derivatives (IC_50_: 12.4–17.9 μm, MTT assay) show higher activity than the parent rapamycin (IC_50_: 18.1 μm) against A549 cells, and compound **73** (Figure 12, IC_50_: 12.8 μm) could induce apoptosis and cell cycle arrest in A549 cells (Huang et al., 2018). Moreover, compound **73** inhibits the phosphorylation of mTOR and its downstream key kinases 4EBP1 and p70S6K1 in A549 cells, revealing that this compound could also display effective inhibitory effect on the mTORC1 signaling pathway as rapamycin. Accordingly, compound **73** has the potential to be developed as a new mTOR inhibitor against lung cancers.

1,2,3-Triazole–containing bergenin derivatives **74** (IC_50_: 1.86–23.05 μm, MTT assay) are endowed with considerable activity against A549 cells, and an SAR study demonstrates that the trifluoromethyl group on the phenyl ring is beneficial for the activity (Kumar et al., 2019). In particular, compound **74a** (IC_50_: 1.86 μm, MTT assay), which is comparable to doxorubicin (IC_50_: 1.98 μm), demonstrates potent activity. Cell cycle analysis show that compound **74a** could induce G2/M phase arrest and lead to the accumulation of cyclin B1 protein. Cell-based tubulin polymeri**z**ation assays and docking studies implies that compound **74a** disrupts tubulin assembly by occupying the colchicine binding pocket of tubulin.

1,2,3-Triazole–containing homoerythrina derivative **75** (IC_50_: 1.89 μm, MTT assay) has excellent inhibitory activity to A549 cells, which is higher than those of pemetrexed (IC_50_: 3.39 μm), rucaparib (IC_50_: 4.91 μm), and harringtonine (IC_50_: 10.55 μm) (Li et al., 2020). 1,2,3-Triazole–containing erythrina derivatives **76a,b** (IC_50_: 0.94 and 0.98 μm, MTT assay) show enhanced activity against A549 cells, and the activity is ∼5 times superior to that of rucaparib (IC_50_: 4.69 μm) (Li et al., 2020). Flow cytometry analysis shows that these hybrids not only significantly arrest the cell cycle in the S phase but also induce the apoptosis of the cells. Further studies suggests that these compounds could inhibit the expression of cyclin A, downregulate the expression of bcl-2/bax, activate caspase-3, and ultimately induce the apoptosis of A549 cells.

A significant part of 1,2,3-triazole–containing methoxygenipin derivatives **77** (IC_50_: 4.53–47.18 μm, SRB assay) are active against A549 cells, whereas the parent genipin (IC_50_: > 20.0 μm) is devoid of activity (Silalai et al., 2020). An SAR study implies that the introduction of dibenzyl ether substituted silyl and long-chain aliphatic groups into the C-10 position of the genipin moiety could increase the activity. Compounds **77a,b** (IC_50_: 4.81 and 4.53 μm) are the most active against A549 cells. Thus, both of them merit further investigations. 1,2,3-Triazole–containing bavachinin derivative **78** (IC_50_: 7.72 μm, SRB assay) is more potent than bavachinin (IC_50_: 30.5 μm) against A549 cells, and a mechanistic study reveals that this compound could induce apoptotic cell death through loss of MMP and PARP cleavage (Gupta et al., 2018). Compound **78** could also inhibit the colony formation, cell migration, and induce the morphological changes concentration-dependently.

1,2,3-Triazole–containing melampomagnolide B derivatives **79** (IC_50_: 1.17–34.13 μm, MTT assay) show considerable activity against A549 cells, and most of them are more potent than the parent melampomagnolide B (IC_50_: 9.94 μm) (Ding et al., 2018). The representative compound **79d** (IC_50_: 1.35 μm) shows significant efficacy by inducing apoptosis, inhibiting the proliferation and migration of cancer cells. Thus, this compound might be considered as a promising anticancer drug candidate for further evaluation. 1,2,3-Triazole–tethered Jiyuan Oridonin A–thiazolin-dione hybrids **80a,b** (IC_50_: 3.5 and 4.8 μm, MTT assay) demonstrates higher activity than Jiyuan Oridonin A (IC_50_: 16.4 μm) against A549 cells, and further mechanistic studies reveals that hybrid **80a** has inhibition on the proliferation of cancer cells by inducing apoptosis and arresting the cell cycle at the G1 phase (Ke et al., 2018).

1,2,3-Triazole–containing ʟ-ascorbic acid derivative **81** (IC_50_: 0.75–9.42 μm, MTT assay) possesses excellent antiproliferative activity against A549 cells, whereas the parent ʟ-ascorbic acid (IC_50_: > 100 μm) is inactive, suggesting that 1,2,3-triazole–containing ʟ-ascorbic acid derivatives may be promising for the discovery of novel anti–lung cancer agents (Harej et al., 2019; Macan et al., 2019).

1,2,3-Triazole–containing phosphonate derivatives **82a,b** (IC_50_: 2.0 and 3.0 μm, MTT assay) show potential activity against A549 cells, and the activity is superior to that of doxorubicin (IC_50_: 4.4 μm), revealing that these compounds warrant further investigations (Makarov et al., 2015). Additionally, the activity of 1,2,3-triazole–containing dibenzo[b, e][1,4]diazepin-11-one hybrid **83** (IC_50_: 0.71 μm, MTT assay) against A549 cells is much higher than that of 5-fluorouracil (IC_50_: 3.47 μm), and this hybrid could induce the G2/M phase of cell cycle arrest and apoptosis, indicating that hybrid **83** could be used for further studies as anti–lung cancer candidates (Praveen Kumar et al., 2016).

Some other compounds which are combined by 1,2,3-triazole and benzodiazepine/benzoxepine/dithiocarbamate/deoxysalinomycin/myrrhanone B /phenanthrene/pyrano(2,3-c)phenazine/paeonol/naphthalimide/sesquiterpene/sulfonate ester/sapinofuranone/triterpene/thiourea/benzothiazinone/benzoxazinone/furan/nimesulide (Kuntala et al., 2015; Poornima et al., 2015; Sudhapriya et al., 2015; Chandrashekhar et al., 2016; Reddy et al., 2016; Shaikh et al., 2016; Abdallah et al., 2017; Battula et al., 2017; Kumar et al., 2017; Lu et al., 2017; Mareddy et al., 2017; Mo et al., 2017; Nguyen et al., 2017; Li et al., 2018; Narsimha et al., 2018; Ou et al., 2019; Yang et al., 2019; Jiang et al., 2020; Kanabar et al., 2020; Madasu et al., 2020; Qi et al., 2020; Zhang et al., 2020) also possess certain activity against lung cancer cell lines, but their activities are generally far inferior to those of the references. Hence, these derivatives still need further structural modifications.

## Conclusions

Lung cancer, as a cancer with the highest morbidity, is the leading cause of cancer-related deaths and has already posed heavy burden on the world health system, which makes an urgent need to develop novel anti–lung cancer agents. 1,2,3-Triazole could readily interact with diverse enzymes and receptors in organisms through weak interaction and has been considered as a privileged structure in medicinal chemistry. 1,2,3-Triazole–containing agent CAI synergizes with sorafenib to combat NSCLC through the inhibition of NANOG and aggravation of apoptosis, indicating that 1,2,3-triazole–containing derivatives are useful scaffolds to develop novel anti–lung cancer agents.

The azide-alkyne cycloaddition reaction is the major strategy to synthesize 1,2,3-triazoles. The thermal Huisgen 1,3-dipolar cycloaddition usually results in poor regioselectivity, but the Huisgen cycloaddition catalyzed by metal catalyst could give excellent regioselectivity. For example, the copper(I)-catalyzed azide-alkyne cycloaddition (CuAAC) usually affords 1,4-disubstituted 1,2,3-triazoles, whereas ruthenium(II)-catalyzed azide-alkyne cycloaddition often provides 1,5-disubstituted triazoles. However, azides are energy-rich and potentially explosive substances, so synthesis of azides should be careful. Even though various 1,2,3-triazoles showed poor solubility, combination of the 1,2,3-triazole moiety with the other pharmocophores demonstrated good soluability, and may solve the issue of solubility.

This review summarizes the current developments, mechanisms of action and SAR of a series of 1,2,3-triazole–containing derivatives (chromene/coumarin, chalcone, 1,2,3-triazole-indole, podophyllotoxin/epipodophyllotoxin, quinoline/quinolone, pyridine, pyrimidine/quinazolinone/nucleoside, and steroid derivatives), and some of them suggest great *in vitro* and *in vivo* efficacy. Overall, rational design of 1,2,3-triazole–containing derivatives may provide novel anticancer agents with excellent potency against various lung cancers, including drug-resistant forms.
